# The associations of oxidative stress and inflammatory markers with obesity in Iranian population: MASHAD cohort study

**DOI:** 10.1186/s12902-024-01590-9

**Published:** 2024-04-29

**Authors:** Hamideh Ghazizadeh, Amin Mansoori, Toktam Sahranavard, Mohamad Nasrabadi, Kaveh Hadiloo, Nazanin Sheikh Andalibi, Marzyeh Azmon, Shima Tavallaei, Ameneh Timar, Gordon A Ferns, Majid Ghayour-Mobarhan

**Affiliations:** 1 CALIPER Program, Division of Clinical Biochemistry, Pediatric Laboratory Medicine, The Hospital for Sick Children, Toronto, ON Canada; 2 Department of Applied Mathematics, School of Mathematical Sciences, Ferdowsi University of Mashhad, Mashhad, Iran; 3 Faculty of Medicine, Mashhad University of Medical Sciences, Mashhad, Iran; 4 Student Research Committee, School of Medicine, Zanjan University in Medical Science, Zanjan, Iran; 5 Metabolic Syndrome Research Center, Mashhad University of Medical Sciences, Mashhad, Iran; 6 Department of Internal Medicine, Faculty of Medicine, Mashhad University of Medical Sciences, Mashhad, Iran; 7 Department of Biochemistry and Nutrition, Faculty of Medicine, Mashhad University of Medical Sciences, Mashhad, Iran; 8 Brighton & Sussex Medical School, Division of Medical Education, Falmer, Brighton, Sussex, BN1 9PH UK

**Keywords:** Obesity, PAB, SOD1, hs-CRP, Uric acid, Body mass index

## Abstract

**Background:**

Low-grade inflammation and stress oxidative condition play a role in the pathogenesis of obesity, and the serum levels of these markers, such as pro-oxidant-antioxidant balance (PAB), high-sensitivity C-reactive protein (hs-CRP), and uric acid may indicate obesity progression. In this study, we aimed to investigate the relationship between obesity with PAB, hs-CRP, and uric acid in the Iranian population.

**Methods:**

This study was derived from the Mashhad Stroke and Heart Atherosclerotic Disorder (MASHAD) study. A total of 7985 subjects aged 35 to 65 years were divided into three groups according to body mass index (BMI) as: normal, overweight and obese groups. Anthropometric indices and biochemical parameters such as PAB, superoxide dismutase type 1 (SOD_1_), hs-CRP, and uric acid were measured in all the participants. We evaluated the association of obesity with inflammatory factors by using multivariate regression analysis. Also, those participants with hypertension, an endocrine disorder, history of cardiovascular diseases and diabetes mellitus were excluded from the study.

**Results:**

There was a positive significant correlation between BMI and serum PAB, hs-CRP and uric acid (*p* < 0.001). While no statistically significant relation was observed between BMI and SOD_1_ (*p* = 0.85). Multivariate regression analysis showed that the risk of overweight and obesity increased 1.02 and 1.03-fold according to increase 10 units of PAB raise in comparison to reference group (normal weight) [(odds ratio (OR): 1.02, 95% CI (1.01–1.03)] and [OR: 1.03, 95% CI (1.01–1.04)], respectively). In addition, hs-CRP serum concentration was significantly associated with a high risk of obesity [(OR: 1.02; 95% CI (1.01–1.03)]. While the high levels of serum uric acid were associated with increased odds of overweight and obesity risk [OR: 1.4; CI (1.39–1.58) and OR: 1.76; CI (1.63–1.89), respectively].

**Conclusions:**

Generally, we showed a significant association between BMI and serum PAB, hs-CRP values and uric acid levels, suggesting the role of these factors as risk stratification factors for obesity.

## Background

Obesity is an important public health challenge worldwide [1] and is related to several disorders including hypertension [2], diabetes [2, 3], cancer [4], and cardiovascular problems [1, 5]. In 2016, the prevalence of obesity was estimated at approximately 650 million adults, 340 million adolescents and 39 million children [6]. A recent systematic review and meta-analysis study indicated that 35.09% of Iranian population are overweight or obese, that was estimated to be 56.55% for people aged over 18 years and 21.11% for people aged ≤ 18 years [7]. Although public knowledge about the harmful effects of obesity has increased in recent years, the mortality rate of this chronic disease is increasing globally [8]. Some risk factors involved in the development of obesity are genetic factors, lifestyle choices and medical conditions [9–11]. Pro-oxidant-antioxidant balance (PAB) is a measure of the status of oxidative stress [12]. Oxidative stress is defined as a condition of imbalance between pro-oxidant and antioxidant production which results in increased reactive oxygen species in tissues [13]. This condition has a major role in the incidence of various diseases such as cardiovascular events [14], diabetes [15] and asthma [16]. The relationship between obesity and oxidative stress is well established; a high consumption of fat and carbohydrates in obese subjects may be associated with an increase in oxidative stress and inflammation [17]. Prolonged obesity is associated with a reduction in the serum activity of antioxidant enzyme including superoxide dismutase type 1 (SOD_1_) and glutathione peroxidase (GPx) [18]. Moreover, there is an association between high levels of serum cholesterol and serum thiobarbituric acid reactive substances (TBARS) with reduced serum anti-oxidants enzymes activities in obese subjects [18].

Prior studies have shown a positive correlation between obesity and the level of high sensitive C-reactive protein (hs-CRP) [19, 20]. It has been reported that oxidative stress has the ability to affect serum hs-CRP and also increases inflammatory processes and markers of atherosclerosis. On the other hand, serum uric acid is associated with oxidative stress [21]. Several studies revealed that serum uric acid has a role in protecting the body from oxidative damage [22, 23]. A positive relationship between serum uric acid and obesity has been previously shown [24–26]. Although there are ample of evidence about the association of body mass index (BMI) and inflammatory/ oxidative stress, up to date, no prior study evaluated these association in a large sample of Iranian general population. Thus, the main aim of the current study is to investigate the association of obesity with biomarkers of stress oxidative and inflammation in Iranian adult population.

## Methods

### Study population

This study was performed on the population of Mashhad Stroke and Heart Atherosclerotic Disorder (MASHAD) cohort study. The MASHAD study was started in 2010 and consists of 9704 participants aged 35–65 years, who were drawn from the north-Khorasan Iran, by a stratified cluster random sampling method (code number: 85,134) [27].

In the current study, we recruited the number of 7985 individuals aged mean 48.68 ± 7.89 years including 3282 men and 4703 women. We excluded all individuals with hypertension, an endocrine disorder, signs of cardiovascular diseases (CVDs) and diabetes mellitus. The same equipment and methods in clinical and laboratory indices were used among participated subjects. Both men and women were divided into four groups according to body mass index (BMI): (a) underweight group; BMI < 18.5 Kg/m^2^, (b) normal group; BMI 18.5–25 Kg/m^2^, (c) overweight group; BMI 25–30 and (d) obese group; BMI > 30 Kg/m^2^ [28]. Participants provided written informed consent to participate in this study, the protocol was previously approved by the Mashhad University of Medical Sciences Ethics Committee. Also, the variable data as past and/or current smoking status, physical activity in daily-time, health behaviors and consumed medications by subjects were obtained by standard questionnaire interviews [27]. The acquired information was recorded and organized to consequence analysis.

### Blood sampling, blood pressure and anthropometric measurements

Blood samples were collected from each participant after 12–14 h fasting into Vacutainer® tubes. The samples were centrifuged for 15 min at 5,000 g to separate the serum which then was aliquoted and kept frozen at -80^o^ C for analysis. Blood pressure (mmHg) was measured twice with each subject being requested to sit and rest for 30 min before each measurement. The two systolic blood pressure (SBP) and diastolic blood pressure (DBP) were recorded and the average was used for the analysis. Height, hip and waist circumference were measured to the nearest 0.1 cm, and weight was measured to the nearest 0.1 Kg without shoes, in fasting state for all individuals, then, BMI was calculated as weight (Kg)/height^2^ (m^2^) [27, 29].

Physical activity level (PAL) determined using the modified standardized questionnaire of the SHHS / MONICA the questionnaire. Questionnaire is about the time the subject spent on activities during work and during the non-work time, as well as resting in bed and sleep. Individuals with 1-1.39 physical activity the level were classified in the inactive group, those who had PAL between 1.4 and 1.59 and 1.6–1.89 were categorized in low activity and moderate activity groups respectively. The PAL of high activity subjects was 1.9–2.5 [30].

### Biochemical analysis and quality control

Lipid profile including serum triglycerides (TG), low-density lipoprotein cholesterol (LDL-C), high-density lipoprotein cholesterol (HDL-C) and total cholesterol and fasting blood glucose (FBG) (mg/dL) were measured as previously reported [27]. The level of serum uric acid (μg/dL) was measured using Pars Azmoon kits on a BT-3000 auto analyzer (Biotechnical, Rome, Italy). Serum hs-CRP levels (mg/L) were determined by polyethylene glycol (PEG-)-enhanced immunoturbidimetry using an Alcyon analyzer (Abbott, Chicago, IL, USA), as described previously [31, 32]. Assayed serum controls at two different concentrations were used to monitor the quality of assays. Intra- and inter-assay coefficients of variation (CV) were both 4.0% for TG, 2.0% for TC, 5% for HDL-C and LDL-C, 2% for FSG, 2.0% for uric acid and 17.0% for hs-CRP.

To SOD_1_ assessment, at first diethylenetriamine pent acetic acid (DTPA) (0.001 M) was added 0.05 M Tris (including 0.001 M DTPA) tocacodylic acid 0.05 M (comprising 0.001 M DTPA) to make Tris-cacodylic acid buffer 0.05 M (pH 8.2) which was then air-balanced for 1 h. Pyrogallol solution (0.02 M) was produced in water which leads to eliminating the soluble oxygen and then was aliquoted and froze until analyzes. Then 20 μl of sample or control was added to wells in duplicate. 0.02 M of pyrogallol stock solution was diluted 1:100 and then 180 μl of the solution was added per well. An enzyme-linked immunosorbent assay (ELISA) reader at 405 nm read the mixture for 1 h at a distance of 5 min. The activity of SOD1 was calculated by the level of SOD_1_ which inhibited the oxidation of pyrogenallol by 50% compared to control.

### PAB assay and quality control

The values of serum PAB were determined and validated as described, previously [14, 33, 34]. Peroxidase enzyme (Applichem, A3791, chemicals used were reagent grade and were collected in double distilled water [35].

3mM uric acid (in 10 mM NaOH) was added into various proportions (0–100%) of 250 μM hydrogen peroxide to prepar the standard solutions. 60 mg of the powder TMB was solved in 10 ml DMSO. TMB/DMSO (400 ml) was added to 20 ml acetoacetate buffer 0.05 M (pH 4.5) after than 70 mL of fresh chloramine T 100 mM was used and the mixture was thoroughly mixed to prepare TMB cation. This mixture was incubated for 2 h at room temperature in the dark place. Then 20 ml of TMB cation and 25 U of peroxidase enzyme solution were mixed which aliquoted in 1 ml and kept at – 20 C. Also, mixing 200 μL of TMB/DMSO with 10 ml acetate buffer 0.05 M (pH 5.8) prepared TMB solution. 1 mL TMB cation into 10 mL TMB solution made the working solution which incubated 2 min at the room temperature in a dark place. Then, 10 ml of standard or any sample was mixed with 200 μL of working solution which then incubated for 12 min in a dark place at 37 ^o^C. Ultimately, in each well of 96 plates well, 100 μL of HCL (2 M) was plated to measure the absorption of the samples using an ELISA reader at 450 nm with a reference wavelength of 620–570 nm. The standard sample for every plate presented the standard curve which used to investigate the amounts of serum samples [36].

For the determination of the precision of the modified PAB method, the intra- and inter-assay coefficients of variation (%CV) were determined. The %CV of the intra-assay for 28 samples analyzed in triplicate was between 1.4 and 3.5%, with a mean of 2.1%. The %CV of the inter-assay for 20 samples, analyzed over 3 days, was between 4.1 and 8.5%, with a mean of 6.1% [34, 37].

### Statistical analysis

The normality of distribution was assessed using the Kolmogorov-Smirnov test. Quantitative data were expressed as the mean ± SD for normally distributed variables or as the median and IQR for not normally distributed variables. For normally distributed variables, the one-way ANOA test and was used. Kruskal-Wallis test was used for non-normally distributed variables. Qualitative variables compared using the Chi-square test. Pearson correlation was used to find a linear relationship between BMI with serum PAB, SOD_1_, hs-CRP, and uric acid. The multivariate regression model was used to assay the association of obesity with serum PAB, SOD_1_, and hs-CRP after adjusting for sex, age, uric acid. *P*-values less than 0.05 were considered significant for all analyses. All statistical analyses were carried out with SPSS (version 20, Chicago, IL, USA).

## Results

The demographic and biochemical characteristics of subjects according to BMI are shown in Table 1 such as waist circumference, hip circumference, weight, SBP, DBP, PAL, FBG, lipid profile, and uric acid levels showed a significant difference between three groups of BMI in both sexes (*P* < 0.001). While no difference was observed for LDL-C, and SOD_1_ between obese and overweight men and women compared to normal individuals. The level of PAB in overweight and obese women was significantly higher than women with normal BMI, however, only obese men showed higher PAB levels compared to overweight and normal groups (*P* < 0.001). Also, the changes in cholesterol levels in three BMI groups were similar in both genders. Furthermore, an increasing trend was observed in the level of TG and hs-CRP in three different BMI groups of both sexes (*P* < 0.001). Also, the smoking category was assessed for both genders which showed no statistical difference between three BMI groups for women (*p* = 0.09), while that was different significantly in normal, overweight, and obese men (*P* < 0.001).

**Table 1:** Baseline characteristics of the participants

Variables	Male				Female			
	Normal (BMI < 25 Kg/m^2^)	Overweight (BMI 25–30 Kg/m^2^)	Obese (BMI > 30 Kg/m^2^)	*P* value	Normal (BMI < 25 Kg/m^2^)	Overweight (BMI 25–30 Kg/m^2^)	Obese (BMI > 30 Kg/m^2^)	*P* value
PAB (HK)	57.09 ± 49.28	59.28 ± 48.16	65.13 ± 49.5 ^b, c^	0.006	70.50 ± 54.68	80.36 ± 59.84 ^a^	80.44 ± 61.81 ^b^	< 0.001
SBP (mmHg)	117.98 ± 16.09	123.73 ± 16.06 ^a^	128.32 ± 18.56 ^b, c^	< 0.001	115.86 ± 17.60	121.23 ± 19.55 ^a^	126.23 ± 19.55 ^b, c^	< 0.001
DBP (mmHg)	76.98 ± 10.22	81.05 ± 10.26 ^a^	84.00 ± 10.70 ^b, c^	< 0.001	74.85 ± 11.03	78.62 ± 11.50 ^a^	81.35 ± 11.23 ^b, c^	< 0.001
SOD_1_(U/L)	2.27 ± 2.35	2.28 ± 2.26	2.24 ± 1.75	0.94	2.34 ± 3.08	2.38 ± 2.84	2.33 ± 2.56	0.88
WC (m)	84.74 ± 8.17	96.10 ± 7.14 ^a^	106.57 ± 8.62 ^b, c^	< 0.001	85.02 ± 9.45	94.12 ± 9.05 ^a^	105.10 ± 10.86 ^b, c^	< 0.001
HC (m)	95.15 ± 5.74	102.35 ± 5.42 ^a^	110.42 ± 7.15 ^b, c^	< 0.001	95.27 ± 5.90	102.98 ± 5.60 ^a^	113.30 ± 8.27 ^b, c^	< 0.001
FBG (mg/dL)	86.95 ± 34.74	93.27 ± 36.74 ^a^	98.06 ± 43.79 ^b, c^	< 0.001	90.58 ± 45.46	95.56 ± 43.94 ^a^	95.04 ± 36.79	0.004
Uric acid (μg/dL)	4.81 ± 1.17	5.47 ± 1.48 ^a^	5.74 ± 1.56 ^b, c^	< 0.001	3.77 ± 1.07	4.20 ± 1.18 ^a^	4.59 ± 1.22 ^b, c^	< 0.001
LDL-C (mg/dL)	112.45 ± 33.34	114.33 ± 35.11	113.08 ± 35.31	0.29	116.44 ± 36.13	120.37 ± 35.80	120.31 ± 36.01	0.007
Cholesterol (mg/dL)	180.49 ± 37.73	189.97 ± 36.98 ^a^	191.61 ± 38.50 ^b^	< 0.001	189.12 ± 40.48	196.28 ± 39.04 ^a^	198.98 ± 40.01 ^b^	< 0.001
HDL-C (mg/dL)	41.61 ± 9.51	38.81 ± 8.85 ^a^	38.69 ± 9.25 ^b^	< 0.001	47.09 ± 10.59	44.92 ± 9.96 ^a^	43.73 ± 9.19 ^b, c^	< 0.001
Weight (Kg)	63.47 ± 7.88	77.75 ± 7.04 ^a^	92.19 ± 10.7 ^b, c^	< 0.001	54.98 ± 6.43	66.76 ± 5.91 ^a^	80.30 ± 9.6 ^b, c^	< 0.001
Height (m)	1.68 ± 0.06	1.68 ± 0.06	1.67 ± 0.07 ^b^	0.003	1.55 ± 0.06	1.55 ± 0.057	1.54 ± 0.05 ^b, c^	< 0.001
PAL	1.5 ± 0.31	1.39 ± 0.25 ^a^	1.27 ± 0.25 ^b, c^	< 0.001	1.85 ± 0.22	1.72 ± 0.19 ^a^	1.58 ± 0.19 ^b, c^	< 0.001
Age (year)	48.25 ± 8.60	49.24 ± 8.38 ^a^	49.11 ± 8.11	0.02	48.33 ± 7.72	48.39 ± 7.52	49.11 ± 7.40 ^b^	0.002
Hs-CRP (mg/L)	1.3 (1.58)	1.44 (1.81)	2.10 (2.99)	< 0.001	1.24 (1.31)	1.68 (2.44)	2.62 (4.29)	< 0.001
TG (mg/dL)	98 (66)	141 (98)	150 (104)	< 0.001	96 (66)	117 (83)	134 (83)	< 0.001
Smoking status				< 0.001				0.09
Non smoker	51.9%	59.9%	62.4%		76.4%	76.9%	73.5%	
Ex-smoker	14.4%	15.3%	16.8%		6.2%	6.6%	6.9%	
Current smoker	33.%	24.8%	20.9%		17.4%	16.6%	19.5%	

Abbreviations: SOD_1_: superoxide dismutase, hs-CRP: high sensitivity C-reactive protein, TG: triglyceride, PAB: pro-oxidant-antioxidant balance, HDL-C: high-density lipoprotein cholesterol; LDL-C: low-density lipoprotein cholesterol; SBP: systolic blood pressure; DBP: diastolic blood pressure; FBG: fasting blood glucose; WC: waist circumference; HC: hip circumference; PAL: physical activity level

Values are expressed as mean ± SD, median and interquartile range for normally and non-normally distributed variables, respectively

a; significant difference between normal and overweight groups

b; significant difference between normal and obese groups

c; significant difference between overweight and obese groups

Regarding to Table 2, BMI had significantly positive correlation with the values of serum PAB (*r* = 0.104, *P* < 0.001), hs-CRP (*r* = 0.156, *P* < 0.001), and uric acid (*r* = 0.134, *p* < 0.001). While no statistical relation was observed between BMI and SOD_1_ (*p* = 0.85). Multivariate regression analysis showed that the risk of overweight and obesity increased 1.02 and 1.03-fold according to increase 10 units of PAB (odds ratio (OR): 1.02, 95% CI (1.01–1.03)) and (OR: 1.03 (95% CI (1.01–1.04)) in comparison to reference group (subjects with normal weight) in Table 3. Also, hs-CRP serum levels were significantly associated with increased odds of overweight and obesity risk (OR: 1.4; CI (1.39–1.58), *P* < 0.001 and 1.76; CI (1.63–1.89), *P* < 0.001, respectively) (Table 3).

**Table 2:** Correlations between BMI and PAB, SOD_1_, hs-CRP, and uric acid

Characteristics	PAB	SOD_1_	hs-CRP	Uric acid
N	7985	6253	7964	7969
r	0.104	-0.002	0.156	0.134
P-value	< 0.001	0.85	< 0.001	< 0.001

Abbreviations: SOD_1_: superoxide dismutase; hs-CRP: high sensitivity C-reactive protein; PAB: pro-oxidant-antioxidant balance.

**Table 3:** Association of obesity with serum PAB, SOD1, hs-CRP, and uric acid

Variables	Overweight (BMI 25–30 Kg/m^2^)				Obese (BMI > 30 Kg/m^2^)			
	B	OR	95% CI	*P* value	B	OR	95% CI	*P* value
PAB^*^(H_2_O_2_)	0.02	1.02	(1.00-1.03)	0.006	0.03	1.03	(1.01–1.04)	0.001
SOD_1_(U/L)	-0.02	0.98	(0.95–1.01)	0.19	− 0.011	0.98	(0.95–1.02)	0.49
hs-CRP (mg/L)	0.00	1.00	(0.99–1.01)	0.41	0.25	1.02	(1.01–1.03)	< 0.001
Uric acid (μg/dL)	0.40	1.4	(1.39–1.58)	< 0.001	0. 56	1.76	(1.63–1.89)	< 0.001

Abbreviations: SOD_1_: superoxide dismutase; hs-CRP: high sensitivity C-reactive protein; PAB: pro-oxidant-antioxidant balance; CI: confidence interval; BMI: body mass index; OR: odds ratio

Multivariate regression model was used and normal weight group is considered as a reference group. Adjusted for sex, age, uric acid

*PAB unit is equal to defined 10 HK

## Discussion

In the present study, for first time, we explored the relationship between BMI and serum levels of both oxidative stress and inflammatory markers in a large sample of Iranian general population. We found that BMI was significantly associated with serum levels of PAB, hs-CRP, and uric acid. Obesity has become an issue among both rural and urban Iranians [38] and is considered as a reason for metabolic syndrome which is correlated to chronic inflammation in obese individuals [9]. In our previous study, we showed that oxidative stress and inflammation are related to the presence of high body fat percentage with the progression of metabolic syndrome, CVD, and diabetes [39]. In this study we used BMI, because it is a cheaper and more available assessment of obesity than body fat percentage.

In obesity status, adipose tissue accumulates and secrets adipokines [40–42] which stimulate the synthesis of pro-inflammatory cytokines and ultimately increase the reactive oxygen species (ROS) generation [43]. ROS production can damage cellular components, leading to inflammation and chronic diseases [44]. An imbalance between production and clearance of ROS (by the antioxidant enzymes), is defined as oxidative stress [45]. The PAB technique can determine the oxidant capacity and assess the status of oxidant/anti-oxidant of a subject in a single assay [46]. Consistent with our results, a study on over-weighted and obese subjects without CVDs, showed that high levels of PAB were observed in obese people (BMI > 30 Kg/m^2^) with mean 40.8 HK/unit, while there was no significant difference between over-weighted (BMI 25–30 Kg/m^2^ ) and normal groups with values of 37.5 and 37.2 HK/unit, respectively [47]. Another study among 338 Iranian population showed that lower pro-oxidants consumption is associated with decreased risk of general and central obesity after adjustment for age and sex [48]. It is also indicated that obese subjects had significantly higher concentrations of PAB rather than overweight in male Iranian adults [49]. However, no significant association was reported between oxidative balanced score and any components of metabolic syndrome in a study on 850 adults living in Tehran, Iran [50]. As studies indicated that increased oxidative stress in adipose tissue is a precursor to metabolic syndrome, balancing of the redox status of adipose tissue may hold promise as a potential treatment target for obesity [51].

It is believed that oxidative stress results from not only ROS overproduction, but also can conclude of disruption in antioxidant enzymatic defenses such as SOD_1_ which converts O_2_^–^ to H_2_O_2_ [52]. Results of a study on mice illustrated that cytosolic SOD_1_ in obese mice was lower than the lean control group [53]. Nevertheless, in our study, the level of SOD_1_ showed no statistical difference between normal and obese or overweight groups in both genders. Additionally, no correlation between BMI and SOD_1_ levels was observed. Although the differences in SOD_1_ concentrations/activities among individuals may be attributable to variants in genes encoding SOD_1_ isozymes [54].

We also showed that the levels of uric acid significantly differs between three groups of BMI in both genders (normal, overweight and obese groups). Also, we showed an association between uric acid and a high risk of overweight and obesity (OR: 1.4% and 1.6%, respectively). A similar study on 550 participants indicated that hyperuricemia was significantly related to obesity in north area of Iran (OR 1.92; 95 per cent CI 1.13, 3.23) [55]. It is revealed that serum levels of uric acid was positively correlated with BMI and waist circumference [56, 57] and according to a 10-year follow-up study, BMI increased by increasing the level of uric acid in both sexes [58]. These findings may be explained by the fact that the obese subjects have an elevated levels of plasma free fatty acids in the liver which stimulates the synthesis of triglycerides and in turn may promote the production of uric acid [59]. Moreover, elevated serum uric acid has deleterious effects on the function of endothelium and oxidative metabolism [60].

In obesity, accumulated free fatty acids (FFAs) can cause pathophysiological mechanisms leading to inflammation and increasing the CRP levels [61]. Our analysis showed a considerable change in hs-CRP levels in obese, overweight compared with normal groups in both sexes. Although there was a significant association between BMI and hs-CRP only in the obese group (*p* < 0.001, OR: 1.02 CI (1.01–1.03)). Similarly, a hospital-based study on 7762 subjects showed that the obese ones have higher levels of hs-CRP [62]. Whereas another study failed to show a significant association between obesity and hs-CRP in 192 Iranian adults [63]. This disparity in results may be due to the different sample size. FFAs are drained by the liver which leads to secret pro-inflammatory adipokines into the portal circulation and promotes interlukine-6 (IL-6) releasing from adipose tissue, which finally results in the secretion of CRP [64, 65]. IL-6 is a mediator of inflammatory process which produces by immune cells and adipose tissue [66]. The high concentrations of IL-6 leads to endothelial/microvascular dysfunction, as well as overproduction of ROS which finally increases the concentrations of systemic CRP [67]. It is reported that each degree of obesity was directly correlated with CRP [68]. As a study on women in Montenegro, the levels of CRP in over-weight women was higher than the normal weight group [69]. According to a study on Indian children, by increasing 1.0 unit of BMI, CRP odds ratios increased by nearly 40% (*p* < 0.001, 95% CI: 1.23–1.53) [70].

Aside from the underlying population-based large scale research’s strengths, a number of limitations need to be noted regarding the current study. First, only participants aged between 35 and 65 years old were evaluated in this study. Second, it is a cross-sectional study, so no causal association could be identified. Third, we have not access to the medications, supplementation, or alcohol history of the participants, so we could not remove the effect of these confounders.

## Conclusion

Generally, this study shows the association between obesity and inflammation and oxidative stress. Understanding of the obesity-associated conditions would be helpful in order to develop new treatments, and for preventing several disorders. Future studies should characterize the potential mechanisms and roles of oxidative stress and inflammation in obesity to control the onset and deterioration of autoimmune and/or inflammatory diseases.

## Data Availability

The datasets used and/or analyzed during the current study are available from the corresponding author on reasonable request.

## Abbreviations

BMI: Body mass index
CV: Coefficients of variation
CVDs: Cardiovascular diseases
DBP: Diastolic blood pressure
FBG: Fasting blood glucose
FFAs: Free fatty acids
GPx: Glutathione peroxidase
HDL-C: High-density lipoprotein cholesterol
hs-CRP: High sensitive C-reactive protein
IL-6: Interlukine-6
LDL-C: Low-density lipoprotein cholesterol
MASHAD: Mashhad Stroke and Heart Atherosclerotic Disorder
PAB: Pro-oxidant-antioxidant balance
PAL: Physical activity level
ROS: Reactive oxygen species
SBP: Systolic blood pressure
SOD_1_: Superoxide dismutase type 1
TBARS: Thiobarbituric acid reactive substances
TG: Triglycerides
